# Novel Desorber for Online Drilling Mud Gas Logging

**DOI:** 10.1155/2016/7014068

**Published:** 2016-03-31

**Authors:** Marcin Lackowski, Marek Tobiszewski, Jacek Namieśnik

**Affiliations:** ^1^The Szewalski Institute of Fluid Flow Machinery, Polish Academy of Sciences, 14 Fiszera Street 14, 80-231 Gdańsk, Poland; ^2^Department of Analytical Chemistry, Chemical Faculty, Gdańsk University of Technology (GUT), 11/12 G. Narutowicza Street, 80-233 Gdańsk, Poland

## Abstract

This work presents the construction solution and experimental results of a novel desorber for online drilling mud gas logging. The traditional desorbers use mechanical mixing of the liquid to stimulate transfer of hydrocarbons to the gaseous phase that is further analyzed. The presented approach is based on transfer of hydrocarbons from the liquid to the gas bubbles flowing through it and further gas analysis. The desorber was checked for gas logging from four different drilling muds collected from Polish boreholes. The results of optimization studies are also presented in this study. The comparison of the novel desorber with a commercial one reveals strong advantages of the novel one. It is characterized by much better hydrocarbons recovery efficiency and allows reaching lower limits of detection of the whole analytical system. The presented desorber seems to be very attractive alternative over widely used mechanical desorbers.

## 1. Introduction

The exploration of energetic resources is to a large extent based on drilling. As the resources become scarcer, the drilling gains importance, as it is required to drill deeper and it is performed in more remote areas. Drilling is important in extraction of natural gas and crude oil. Recently unconventional gas resources such as shale gas [1] (from shale rocks formations) or tight gas [2] (from pores of rocks, mainly sandstones) gained much interest.

There are two main reasons to pump water to the borehole. The first one is to lubricate and cool the drill. The other reason is to remove rock cuttings together with water flowing out of borehole. Drilling liquid, often called drilling mud, is heavy liquid consisting of water with rock cuttings [3]. Drilling mud is analyzed for dissolved gasses and other chemical parameters, which is the convenient way to give the information about the rock formations at the level of drilling bit. The gases are released from rock as the drill bit mechanically releases them from rock pores. The gas is routinely analyzed for hydrocarbons; either total concentration of C_1_–C_5_ alkanes or these compounds are determined separately. Real time data plot for the drilling time is an important document for the interpretation of the structure of the rock formations. Mud gas logging is widely accepted as routine way to obtain important geochemical information.

Apart from great importance for industrial drilling, real time drilling fluids analysis is performed for scientific reasons. The determination of He and Rn in drilling fluids can give information about the tectonic stability after an earthquake [4]. Similarly, drilling fluids analysis gives precious information about magma evolution processes in the neighborhood of active volcano [5].

The aim of this work is to present the novel gas desorber applied for the analysis of dissolved gases in drilling mud. The construction solution and its performance in recovery of dissolved methane are presented.

## 2. Materials and Methods

### 2.1. Novel Gas Desorber

**Figure 1:**
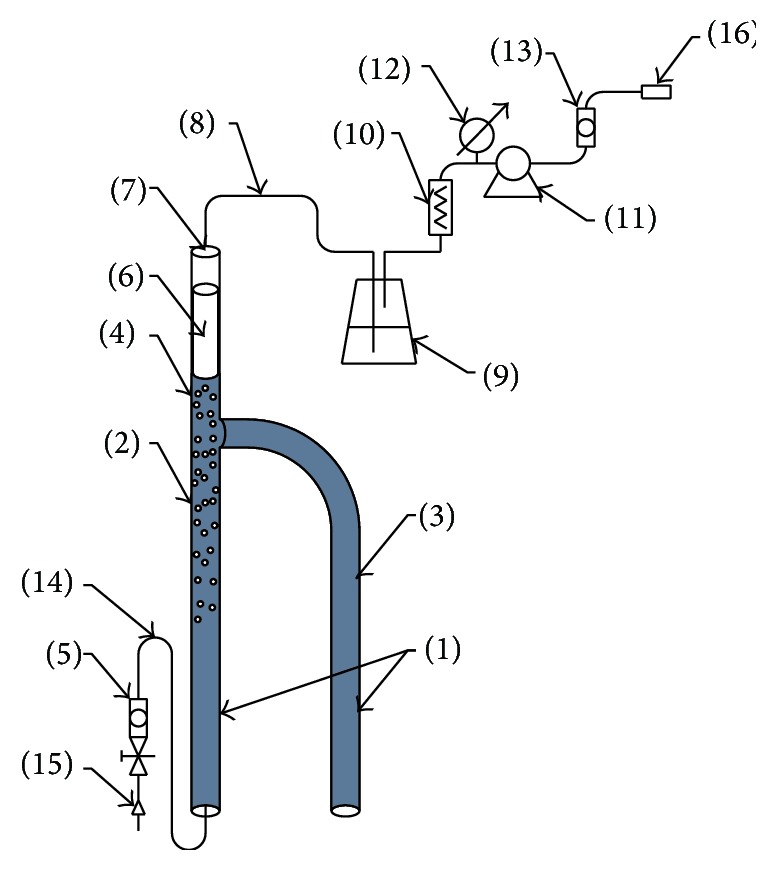
The scheme of the novel gas desorber.

The construction solution of the novel gas desorber with accompanying system is presented in Figure 1. The desorber (1) consists of the proper chamber (2) and the side arm (3), which is opened at its bottom. The proper chamber is equipped with upper arm (4), which is closed from the upper part. The inert gas inlet line (14) is connected to the bottom of the proper chamber (2), while float (6) and jet (7) are placed in the upper arm. The jet is an outlet (8) for the gases, which is connected through the condensate separator (9), particulate matter filter (10), and pump (11) with analytical device (16). Between the outlet of gases and the pump, there are a vacuum meter (12) and flow meter (13); another flow meter (5) is placed at the gas inlet (15). In order to collect the sample of gas dissolved in the liquid chamber, an appropriate side arm of the desorber liquid is immersed. As a result of the impact of the vacuum produced by the apparatus liquid is sucked into the right side arm. The transfer of the gases contained in the liquid to the air bubbles takes place, and the formed gas mixture is directed to the analytical device. The liquid contained in the side arm contains no gas bubbles; therefore its density is greater than the density of the liquid in the proper chamber. This causes the circulation of the liquid through the desorber and spontaneously determines the ratio of liquid treated to the amount of gas being desorbed from the liquid. Thanks to the novel desorber construction, a significantly greater recovery of dissolved gases is provided from drilling fluids, thereby providing more accurate information about the deposits.

### 2.2. Typical Gas Desorbers

**Figure 2:**
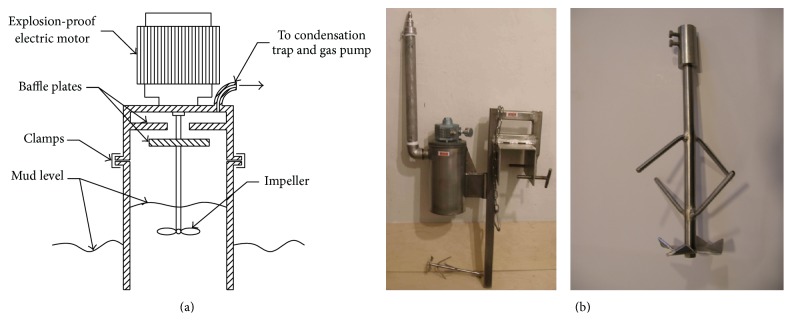
The classical desorber: (a) scheme of operations; (b) picture of the desorber (left) and the mixer (right).

One of the main parts of the online drilling mud gas logging is the gas desorber as it is indicated in Figure 2. The role of the desorber is to remove dissolved gases from the liquid. The typically used desorber consists of a main chamber opened at the bottom, provided with air inlet duct and an outlet duct gases, which is connected to the pump via the condensate separator. In order to collect the sample of gases dissolved in the drilling mud the analytical device chamber is immersed in the liquid, and the mixer is turned on, so that mixing takes place in an intensive manner. In the headspace of desorber chamber, which is not immersed in the liquid, small droplets are formed. After starting the gas suction, liquid droplets are in contact with the outside air. As a result of contact with the air, desorption of gas contained in the liquid droplets takes place. A disadvantage of this system is that only a small fraction of the gases dissolved in the mud is released in the desorber and passes through the inlet with intake gas. At low concentrations of gases in drilling mud measurements are unreliable and subject to considerable errors.

### 2.3. The Experimental System

To perform characterization of the novel desorber, the experimental station was designed. It consists of measurement chamber, where the working fluid (water or drilling mud) was placed, the gas saturation system, allowing obtaining the conditions similar to in-field ones, and the transfer system allowing moving drilling fluid from saturation to measurement zone. Additionally, the experimental station was equipped with flow meters and data acquisition system, the self-constructed C_1_-C_2_ detection system. The detection system is based on flame ionization detector and signal amplifier. All the parts were integrated in single measuring system and were designed to be able to work in field (the real boreholes). It was particularly important to assure adequate isolation and operational reliability of electronic elements of the system.

**Figure 3:**
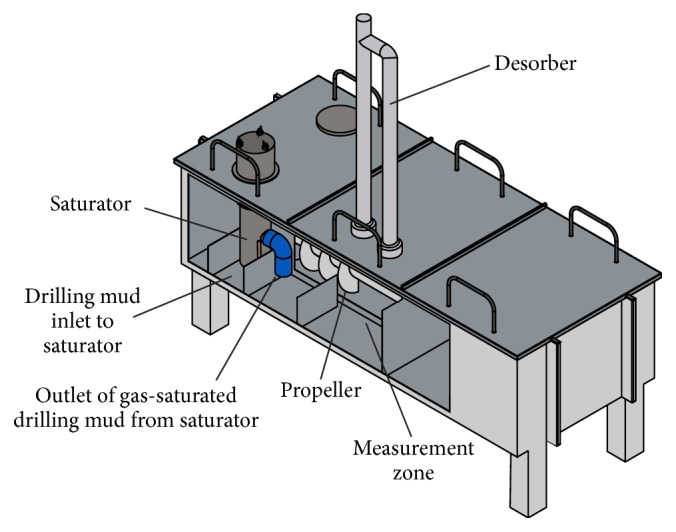
The scheme showing the main parts of the experimental system.

The scheme of the experimental system is presented in Figure 3. The cross section of measurement chamber shows the system assuring the flow of drilling mud between gassing area and measurement area and the location of barriers. The drilling mud or other liquid that is present in the measurement chamber fills the volume between barriers. The propeller is immersed in the liquid and it assures the movement of the liquid between saturation and measurement zones in such a way that no mixing of saturated and degassed liquid streams is possible. The propeller was electric engine-operated, where rotations were optimized to obtain desired flow velocity of liquid in the chamber. The steering of rotation speed of the engine was performed with the inverter. The measurement chamber was tightly closed with covers designed in such a way to allow ease assembly and disassembly of single system parts, like saturator and desorber without the necessity to remove other parts. The surface of the chamber edge was lined with the rubber seal.

**Figure 4:**
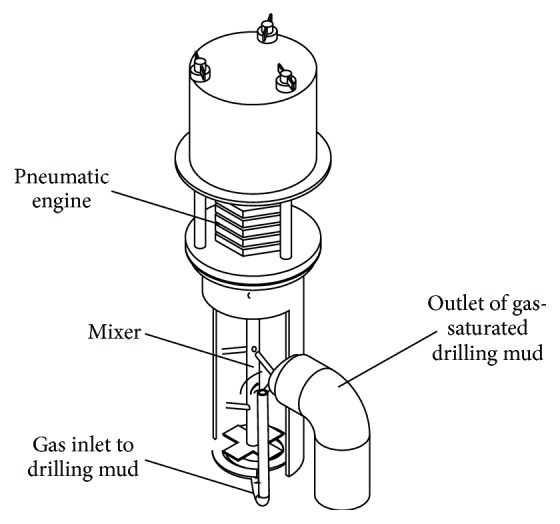
The scheme showing the gas saturation system.

One of the most important parts of the experimental setup was the saturation system allowing introducing gas into liquid. This system was constructed as a part of the project. It utilizes the rotary movement of the agitator that breaks the structure of the liquid with simultaneous controlled delivery of the gas. The gas delivered to the saturator in this project was the mixture of the air and methane containing 0.5, 1, or 2% of methane. The system of gaseous mixture preparation was also designed as a part of this project and is presented in Figure 4. The liquid containing gas is introduced to the chamber that is partially separated with the barrier. It prevented mixing of liquid saturated and not saturated with gas. After gas saturation the liquid is introduced to the desorber zone with forced movement caused by the propeller. Important is the location of the propeller, in such a way that its work does not cause degassing of the liquid; it is located in parallel to the saturated liquid and is separated with a barrier. The propeller is operated with pneumatic engine supplied with compressor (Atlas Copco, Sweden).

The experimental system was equipped with flow meters with controllers placed at the input desorber line and gaseous mixture to the saturator. The underpressure was also controlled and it was generated between desorber and the system of hydrocarbons determination. The other important part was ultrasound flow meter located at the arm outlet of the desorber. During the operation of the desorber the liquid flow velocity is relatively small, which is related to the character of the forcing flow in the arms of the desorber. This is the reason why such system, influencing the geometry of canals, was applied. The ultrasound flow meter FLEXIM FLUXUS ADM with GLQ probes was applied.

All the system elements were integrated in single measurement system based on platform National Instruments. For the data acquisition NI PCI-6281 card was used that was installed on the PC and the module measuring system NI SCXI with dedicated modules for signals conditioning was used. The system was equipped with the set of sensors and converters. The measurement system was equipped with dedicated software, based on the environment NI LabVIEW. The created software allows for online monitoring of drilling fluids at boreholes. The system allows for the measurements of liquid flow, pressure, and the concentration of hydrocarbons in the drilling mud.

## 3. Results and Discussion 

### 3.1. Modeling of Methane Transfer in Water: Air Bubbles System

The main reason of light hydrocarbons exhalation from drilling mud in the desorber is the occurrence of physicochemical nonequilibrium between gaseous and dissolved hydrocarbons. In the presented desorber the liquid-gas interphase surface exists in the form of air bubbles and the upper surface of liquid in desorber column. During short period of time after starting the desorber operation, the air above the column is pushed out by gas from the bubbles that contain hydrocarbons. It is expected that desorption from the surface of column stops and the only carrier of desorbed hydrocarbons will be the bubbles.

It should be noted that the reason for desorption could be also the change of hydrostatic pressure in the drilling mud column. However, the desorption due to this phenomenon would occur only for solution close to liquid saturation. If the desorber has to operate as the sensor informing about drilling bit approaching the gas-containing rock deposits, it would operate in solutions far from being saturated. The abovementioned mechanism of desorption would not have significant influence on its operation.

In the light of these considerations, only the analysis of desorption kinetics to air bubbles will be performed in this work. Because of the complexity of the phenomenon, it was assumed that the dynamics of hydrocarbons desorption in the desorber can be described by the analysis of methane behavior. Moreover the drilling mud was substituted with water. Water is the base for the most of the drilling muds. The crucial issue is determination if the concentration of desorbed methane in the bubbles is close to equilibrium.

To describe methane desorption kinetics from water to the bubble of air the model was selected, in which the rate of desorption is proportional to the difference in concentration of this gas in both phases. The methane transferred from water to the bubble of air can be described by the equation [6–9]:

(1) $$\begin{array}{c} \frac{dN}{dt}=\alpha A_{g}\left(\;c_{g}^{*}-c_{g}\left(\;t\;\right)\;\right). \end{array}$$

In this equation *N* stands for the number of moles of methane transferred through interphase surface. The changes of *N* values in time *t* depend on the difference between actual molar concentration of methane in the bubble *c*_*g*_ and the equilibrium concentration *c*_*g*_^*∗*^. It is proportional to the interphase surface area (of the bubble) *A*_*g*_ and total mass transfer coefficient *α*.

In the solutions with low concentrations the equilibrium ratio between the concentrations of solute in liquid and gas is constant. The value of this concentration ratio is called Henry's constant or equilibrium constant *K*:

(2) $$\begin{array}{c} K=\frac{c_{g}^{*}}{c_{L}}. \end{array}$$

In this case *c*_*L*_ is molar methane concentration in liquid or water. Because the volume of water phase is much larger than the volume of bubbles in desorber column, it was assumed that the value of *c*_*L*_ is constant along the height of column. The value of *K* equilibrium constant is described by the relation:

(3) $$\begin{array}{c} K=\frac{1}{HRT}, \end{array}$$

where *R* is universal gas constant and *T* is absolute temperature, in which the dissolution of solute occurs (desorption). *H* is proportionality constant from Henry's law, defining the relation between the equilibrium proportionality constant of solute *c*_CH_4__ and its partial pressure *p*_CH_4__:

(4) $$\begin{array}{c} H=\frac{c_{CH_{4}}}{p_{CH_{4}}}. \end{array}$$

The value of equilibrium constant *H* is determined experimentally for a given solute (in this case methane) [10].

The change *dN* of methane moles number can be described as the change of its concentration according to the relation:

(5) $$\begin{array}{c} dN=V_{g}dc_{g}, \end{array}$$

where *V*_*g*_ is the volume of bubble.

After substitution of (2) and (5), the kinetic equation (1) can be presented in the form

(6) $$\begin{array}{c} \frac{dc_{g}}{dt}=\alpha a_{g}\left(\;Kc_{L}-c_{g}\;\right), \end{array}$$

in which *a*_*g*_ means the density of interphase surface, defined as

(7) $$\begin{array}{c} a_{g}=\frac{A_{g}}{V_{g}}. \end{array}$$

For sphere-shaped bubbles the function is relatively simple:

(8) $$\begin{array}{c} a_{g}=\frac{6}{D_{g}}, \end{array}$$

negatively proportional to the diameter of the bubble *D*_*g*_. Due to small hydrostatic pressure changes in the desorber column it was assumed that bubble diameter is constant during whole duration of desorption process.

The total mass transfer coefficient *α* is described by the equation

(9) $$\begin{array}{c} \frac{1}{\alpha }=\frac{K}{k_{L}}+\frac{1}{k_{g}}, \end{array}$$

which connects mass transfer with liquid *k*_*L*_ and gas (air) *k*_*g*_ coefficients. Their values are determined experimentally [12–14].

After integration of kinetics equation (6) the relation of methane concentration changes in the bubble in the time function:

(10) $$\begin{array}{c} c_{g}\left(\;t\;\right)=Kc_{L}\left[\;1-\exp\left(\;-\frac{6\alpha }{D_{g}}t\;\right)\;\right]. \end{array}$$

Because it is required to know the kinetics of methane reaching the equilibrium state in the bubble, it is useful to calculate the relative concentration:

(11) $$\begin{array}{c} {\overset{-}{c}}_{g}=\frac{c_{g}}{c_{g}^{*}}\times 100\%, \end{array}$$

which reaches the value of 100% in the equilibrium conditions, when the methane concentration is in maximum. Applying (2) and (10), from definition (11) the following equation can be derived:

(12) $$\begin{array}{c} {\overset{-}{c}}_{g}\left(\;t\;\right)=\left[\;1-\exp\left(\;-\frac{6\alpha }{D_{g}}t\;\right)\;\right]\times 100\%. \end{array}$$

From the properties of exponential function it can be concluded that the time of reaching 100% saturation is infinitely long. That is why it is assumed, for practical purpose, that maximum relative concentration would be ${\overset{-}{c}}_{g}=99\%$. According to (12), the desorption time needed to reach this value will be

(13) $$\begin{array}{c} t_{99}=-\ln\left(\;0.01\;\right)\frac{D_{g}}{6\alpha }\approx 0.77\frac{D_{g}}{\alpha }. \end{array}$$

The desorption time described by (13) can be applied to design the height of desorber column. For this purpose it is additionally required to know the velocity of the bubbles traveling through column due to buoyancy force.

The sample desorption kinetics calculations were done for the proposed desorber, in which column was 50 mm in diameter and 1300 mm in height. The calculations were done for different values of bubble diameter *D*_*g*_ in the range from 0.5 to 2.5 mm. To assess the path that bubble travels before relative methane concentration reaches the value ${\overset{-}{c}}_{g}=99\%$, the equation describing the velocity of bubble was applied [15]:

(14) $$\begin{array}{c} w_{g}=\sqrt{\frac{4D_{g}g\left(1-\rho_{g}/\rho_{L}\right)}{3C_{D}},} \end{array}$$

which can be applied for the air bubbles in diameter *D*_*g*_ < 2.6 mm. In (14), *g* means gravity, *ρ*_*g*_ and *ρ*_*L*_ are the densities of air and water, respectively, and *C*_*D*_ is the resistance coefficient:

(15) $$\begin{array}{c} C_{D}=\frac{24}{Re}+\frac{3}{\sqrt{Re}}+0.34. \end{array}$$

Dependent on Reynolds number,

(16) $$\begin{array}{c} Re=\frac{D_{g}w_{g}\rho_{L}}{\mu_{L}}, \end{array}$$

in which *ρ*_*L*_ is the dynamic viscosity of water. Reynolds number depends also on the unknown velocity *w*_*g*_, so (14)–(16) have to be solved together with the iterative method. The velocity of the bubbles *w*_*g*_ increases almost linearly with the increase of their diameter, but at the diameter about *D*_*g*_ = 2.5 mm significant decrease of the velocity change is observed, which is shown in the following paper [16].

To determine the total mass transfer coefficient *α* (9) it is needed to know the values of permeation coefficients *k*_*L*_ and *k*_*g*_. Coefficient *k*_*L*_ was determined from the relation [13]:

(17) $$\begin{array}{c} k_{L}=0.0113\sqrt{\frac{w_{g}d_{{CH}_{4}}}{0.45+0.2D_{g}}}\;\left[\;{m\;s}^{-1}\;\right], \end{array}$$

where velocity *w*_*g*_ has to be introduced in [cm s^−1^], coefficient describing methane molecular diffusion in water *d*_CH_4__ in [cm^2^ s^−1^], and bubble radius *D*_*g*_ in [cm]. Diffusivity *d*_CH_4__ was determined from the relation [16]:

(18) $$\begin{array}{c} d_{CH_{4}}=\frac{13.26\cdot 10^{-5}}{\mu_{CH_{4}}^{1.14}V_{CH_{4}}^{0.589}}\;\left[\;{cm}^{2}\;s^{-1}\;\right], \end{array}$$

where *μ*_CH_4__ is methane viscosity [cP] and *V*_CH_4__ its molar volume [cm^3^ mol^−1^].

The value of permeation coefficient *k*_*g*_ depends also on the size of bubble and according to the literature [13, 14] it is practically independent of the type of the dissolved gas. For small bubbles, characterized by the diameter *D*_*g*_ = 0.35 mm it is 38.3 cm s^−1^ and decreases to 3.2 cm s^−1^ for *D*_*g*_ = 4.2 mm [6]. For methane it does not have significant influence on the value of total coefficient *α*, because *H*/*k*_*L*_ ≫ 1/*k*_*g*_.

**Figure 5:**
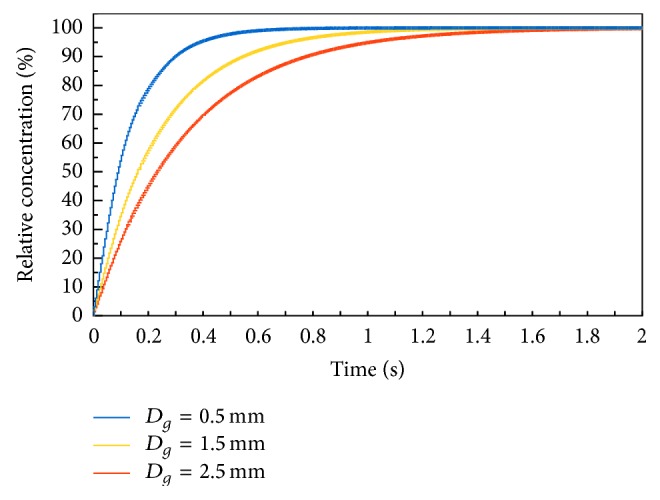
Changes of relative methane concentration *c*_*g*_ in bubbles of diameter *D*_*g*_ equal to 0.5, 1.5, and 2.5 mm.

**Figure 6:**
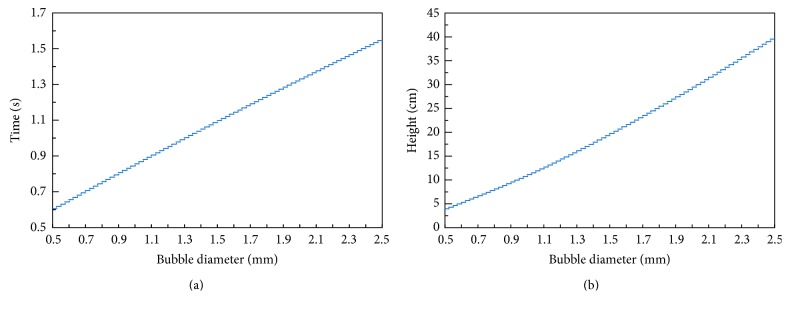
Time and column height needed for relative methane concentration reaching *c*_*g*_ = 99%, in the function of diameter *D*_*g*_.

Table 1 summarizes the values of physical and thermodynamic parameters that were used in the calculations of methane desorption kinetics. With these parameters the changes of relative methane concentrations in air ${\overset{-}{c}}_{g}$ (12), time *t*_99_ (13), and corresponding column height needed to reach relative saturation of 99% were calculated. The results are presented in Figures 5 and 6.

From the performed calculations it can be concluded that the saturation of air bubbles with methane is relatively fast process. For the bubbles of *D*_*g*_ = 0.5 mm reaching the relative methane concentration *c*_*g*_ = 99% takes only *t*_99_ = 0.6 s, with bubbles velocity of *w*_*g*_ = 6.3 cm s^−1^; it is reached on pathway equal to 3.8 cm. With the increase of bubble diameter the time and the pathway grow linearly to 1.55 s and 39.8 cm for the diameter *D*_*g*_ = 2.5 mm. The total path that bubbles travel in the desorber is 130 cm, so it is considerably larger from these values. It can be concluded that the desorber allows obtaining air saturated with methane during sample collection.

### 3.2. Optimization the Desorber Operating Conditions

The novel desorber was examined with above described experimental conditions for the real drilling muds collected from boreholes located in Poland. The drilling muds had the densities between 1.08 kg dm^−3^ and 2.35 kg dm^−3^ (please see Table 2) and were collected from drilling sites Exallo Drilling, which is property of PGNiG company, the largest polish company in the energetic sector. The selection of such different drilling muds was aimed at checking the performance of the novel desorber in the different conditions that can be met in field.

**Figure 7:**
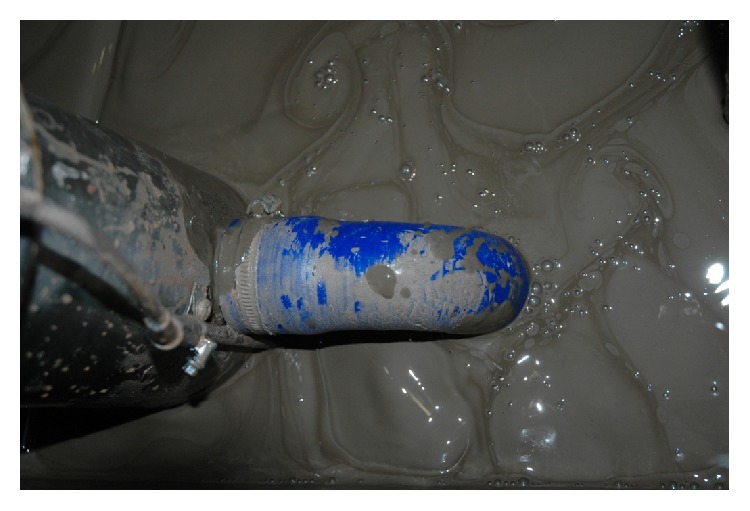
The picture of drilling mud containing gas, drilling mud from Sowia Góra borehole.

The measurements of the desorber performance were done for all the drilling muds described in Table 2. During measurement the gas saturator system was used that allowed obtaining the desired concentration of methane in the drilling mud before the measurement with the novel desorber.  Figure 7 shows the picture of gas-containing drilling mud. Small gas bubbles shown in the picture are characteristic for the drilling muds that are analyzed in real boreholes.

During the process of drilling mud saturation with air and methane mixture the gas was introduced in accurate manner to represent the real drilling mud that is coming out from borehole during drilling process. The drilling mud was subject to desorption with the novel, presented in this study desorber as well as commercially available desorber of PET 21 type in the laboratory. During in-field studies the desorber was also compared with widely applied in Poland desorber of QGM Degasser type. Both desorbers that were compared to the novel in this study are based on the technique of mechanical mixing to destroy the structure of the drilling mud and to recover gas contained in the drilling mud in form of bubbles as well as dissolved in the liquid itself. The construction developed in this project is characterized by different approach; according to our best knowledge it is not applied in any other available desorbers.

**Figure 8:**
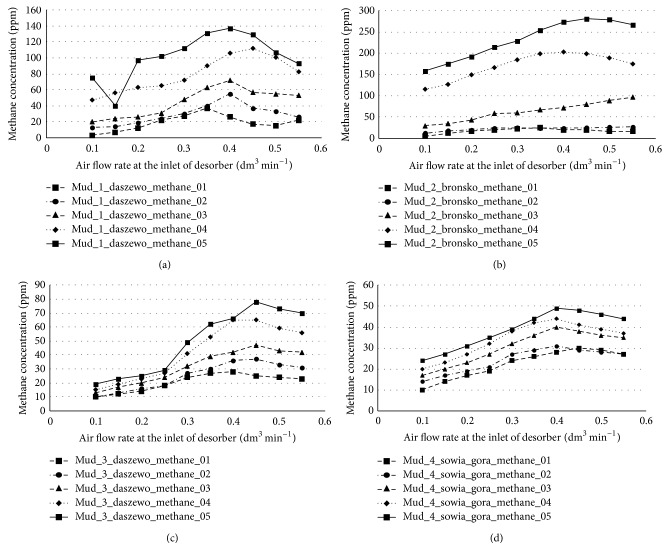
The gas concentration of methane in the function of air flow (dm^3^ min^−1^) at the inlet of the desorber for different methane saturation of drilling mud from (a) Daszewo borehole (density 2.06 kg dm^−3^), (b) Brońsko borehole (1.67 kg dm^−3^), (c) Daszewo borehole (1.18 kg dm^−3^), and (d) Sowia Góra borehole (1.08 kg dm^−3^).

During the experiments one of the important parameters was the amount of air at the input of desorber dependence on the efficiency of desorber (or sensitivity of the system). To optimize this parameter the series of experiments were performed, where the flow intensity was changed for different methane concentrations in the drilling muds and for different types of drilling muds themselves. The justification for these measurements was the great variability of parameters of drilling muds that are used in polish drilling-based mining. The drilling muds depending on their composition and density to different extent “hold” and transport the gas in form of bubbles. Figures 8(a) and 8(b) show the concentration of methane, measured with the presented system, as a function of air flow at the desorber inlet for the drilling mud from Daszewo (density 2.06 kg dm^−3^) borehole and from Bronsko borehole (density 1.67 kg dm^−3^), respectively.  Figure 8(c) shows the results for Daszewo borehole (density 1.18 kg dm^−3^) and Figure 8(d) shows the results for Sowia Góra borehole (density 1.08 kg dm^−3^).

The results presented in Figure 8 allow selecting the optimal air flow rate at desorber inlet to maximize the sensitivity of the measuring system. The highest sensitivity of the system was obtained for the air flow intensity of 0.35 L min^−1^. For lower air flow rates the recovery of methane was smaller, while higher air flow rates caused dilution of methane in the excessive amount of air.

### 3.3. Comparison with Commercial Desorber

**Figure 9:**
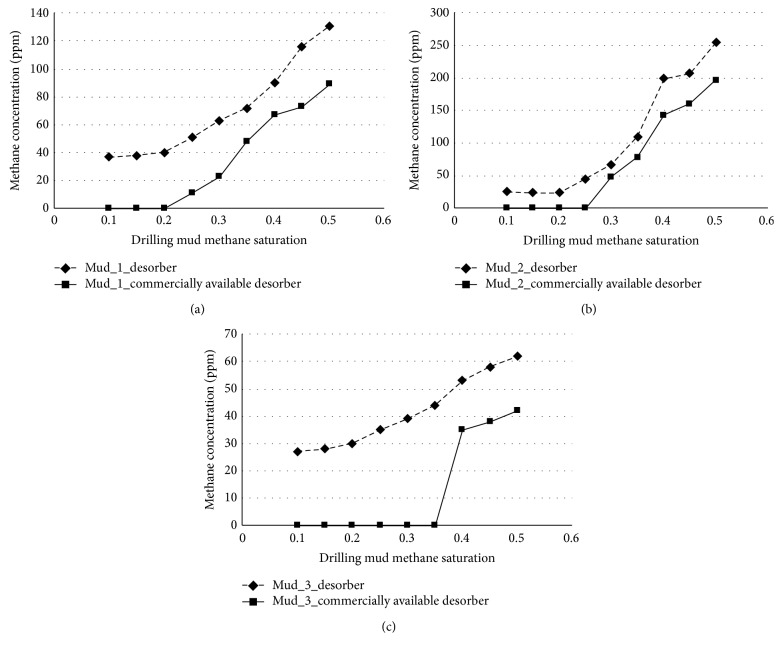
The measured concentration of methane in the function of drilling mud methane saturation for novel and commercially available desorbers. The results for (a) heavy drilling mud from Daszewo borehole (density 2.06 kg dm^−3^), (b) medium density drilling mud from Brońsko borehole (density 1.67 kg dm^−3^), and (c) light drilling mud from Daszewo borehole (density 1.18 kg dm^−3^).

The next part of the study was the comparison of the novel desorber performance with commercially available one (based on the stirrer). During this study the experimental stadion show in Figure 9 was used. The procedure was based on several steps. In the first step in the measurement chamber the novel desorber was placed and connected to TotalGas analyzer. The drilling mud in measurement chamber was saturated with air-methane mixture and with previously presented drilling mud transfer system it was transferred to the measurement zone. The saturation parameters and desorber operational parameters were variables in this experiment. Then, the novel desorber was replaced with commercially available PET 2 desorber. This model was selected because it is characterized with similar size, which was important to ensure comparable operational conditions. The desorber QGM Degasser, more frequently used in the drilling industry, was not selected for the comparison studies. It is characterized by larger size, larger amount of drilling mud required for operation, and its larger flow rates, so the comparison with novel desorber was not possible in similar experimental conditions.

The comparison studies results for different drilling muds are presented in Figure 9(a) for Daszewo borehole drilling mud (density 2.06 kg dm^−3^), in Figure 9(b) for Brońsko borehole drilling mud (density 1.67 kg dm^−3^), and in Figure 9(c) for Daszewo borehole drilling mud (density 1.18 kg dm^−3^).

The results presented in Figure 9 show a better performance for the novel desorber. For all three drilling muds the measured methane concentrations are higher for novel desorber for the same experimental conditions. This shows that the methane recovery from drilling mud is higher for the novel desorber. For all three drilling muds the difference in measured gaseous methane concentrations is 20–30 ppm. The commercially available desorber gives higher limits of detection; the lowest concentration values obtained depend on the drilling mud and are within range of 20–50 ppm. Both better parameters prove that hydrocarbon desorption is more efficient when gas desorption is applied over mechanical mixing as desorption mechanism. The results prove that the novel desorber has potential in-field application.

## 4. Conclusions

The novel desorber for on-line drilling mud logging was presented in detail. The numerical analyses of the desorption process in the presented desorber construction were shown in the paper, too. In contrary to conventional ones that are based on mechanical mixing of the liquid, the novel desorber operation is based on gas flow through the liquid. The results show that the optimal gas flow rate is 0.35 L min^−1^ for the drilling muds collected from different real boreholes. The comparison of the novel desorber with commercially available one shows that novel one is more efficient in recovery of hydrocarbons from liquid. The novel desorber, in authors' opinion, is very attractive device to be applied in field in online drilling mud hydrocarbon or other gases logging. The original desorber, described in the paper, is applied during drilling activities in Poland.

## Figures and Tables

**Table 1:** Physical and thermodynamic parameters used in calculations.

Parameter	Symbol	Unit	Value
Temperature	*T*	K	293
Pressure	*p*	Pa	10^5^
Gas constant	*R*	J mol^−1^·K^−1^	8.31446218
Methane molar mass [11]	*M*_CH_4__	g mol^−1^	16.0425
Methane Henry constant [10]	*H*	M atm^−1^	0.0014
		mol m^−3^·Pa^−1^	1.382 · 10^−5^
Water density [11]	*ρ*_*L*_	kg m^−3^	998
Methane density [11]	*ρ*_CH_4__	kg m^−3^	0.65941
Water dynamic viscosity [11]	*µ*_*L*_	cP = mPa s	1.0016
Methane dynamic viscosity [11]	*µ*_CH_4__	cP = mPa s	0.011024
Methane molar volume [11]	*V*_CH_4__	dm^3^ mol^−1^	24.341
Gravitational acceleration	*g*	m s^−2^	9.81

**Table 2:** The densities of drilling muds originating from different boreholes.

Name of borehole	Drilling mud density [kg dm^−3^]
Sowia Góra	1.08
Daszewo	1.18
Brońsko	1.67
Daszewo	2.06
